# Supplementary material for: Visible-Light Activation of Photocatalytic for Reduction of Nitrogen to Ammonia by Introducing Impurity Defect Levels into Nanocrystalline Diamond
Source: Materials (Basel). 2020 Oct 14;13(20):4559. doi: 10.3390/ma13204559 (PMC7602236; doi:10.3390/ma13204559)
Supplement: Supplementary file 1 [file materials-13-04559-s001.zip › materials-947233-supplementary.docx]

Supplementary Materials

Visible-Light Activation of Photocatalytic for Reduction of Nitrogen to Ammonia by Introducing Impurity Defect Levels into Nanocrystalline Diamond

Rui Su, Zhangcheng Liu, Haris Naeem Abbasi, Jinjia Wei and Hongxing Wang *

Key Lab for Physical Electronics and Devices, Faculty of Electronics and Information Engineering, Xi’an Jiaotong University, Xi’an 710049, China; angelina123@stu.xjtu.edu.cn (R.S.); zcliu_2043@xjtu.edu.cn (Z.L.); haris1996@stu.xjtu.edu.cn (H.N.A.); jjwei@mail.xjtu.edu.cn (J.W.)

***** Correspondence: hxwangcn@mail.xjtu.edu.cn


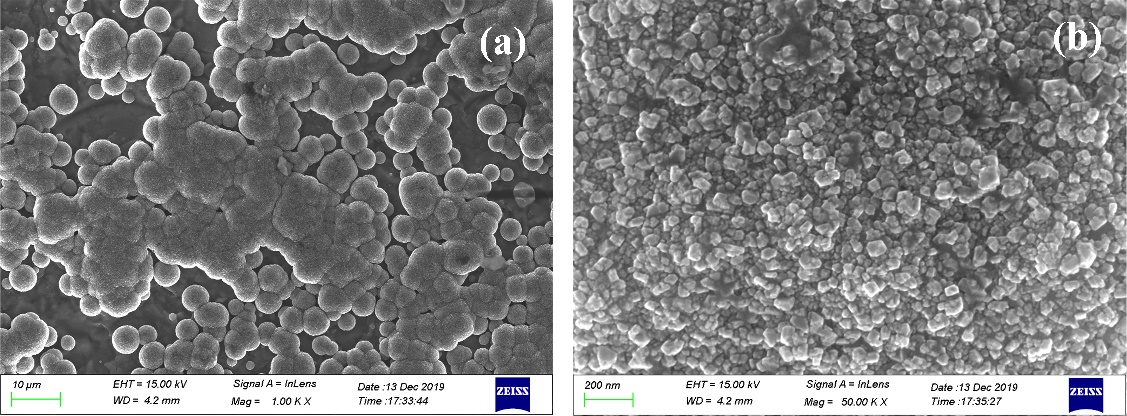


**Figure S1.** SEM images of nitrogen-doped diamond film at different scales.


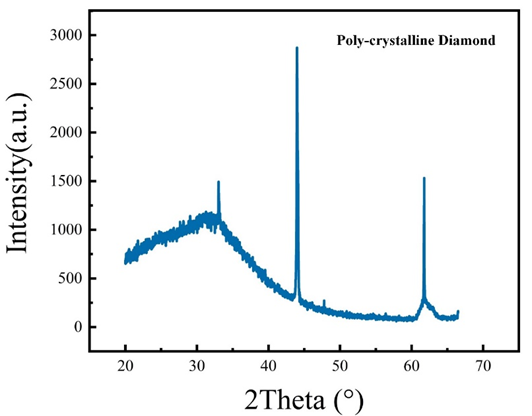


**Figure S2.** XRD spectrum of polycrystalline diamond, different crystal plane orientations correspond to diffraction angles.


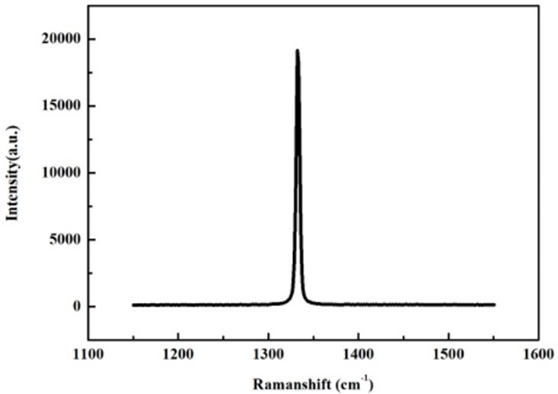


**Figure S3.** Raman spectrum of single crystal diamond, the peak position of is 1332.15 cm^-1^, which is the characteristic peak position of single crystal diamond.


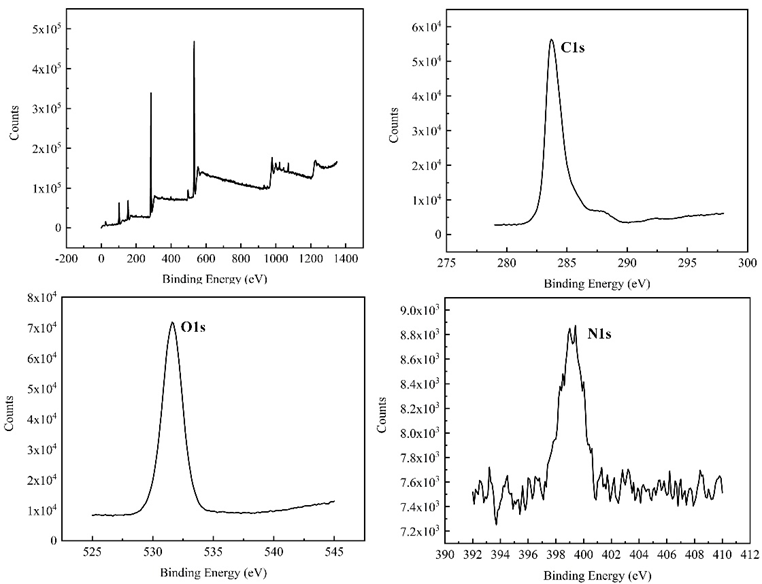


**Figure S4.** the XPS spectrum of nitrogen doped diamond after nitrogen reduction, the high-resolution spectra of the C1s, O1s, and N are measured, since XPS is an important surface analysis technique providing surface information with a thickness of 3–5 nm, we pay more attention on the change on surface of C–O before and after experiments.


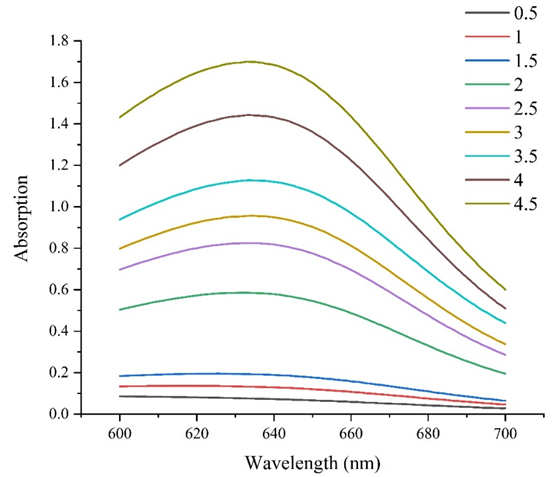


**Figure S5.** a standard absorption curve was obtained using ammonium chloride solutions with known concentration by indophenol blue method. The formation of indophenol blue was determined using absorbance at a wavelength of 635 nm.


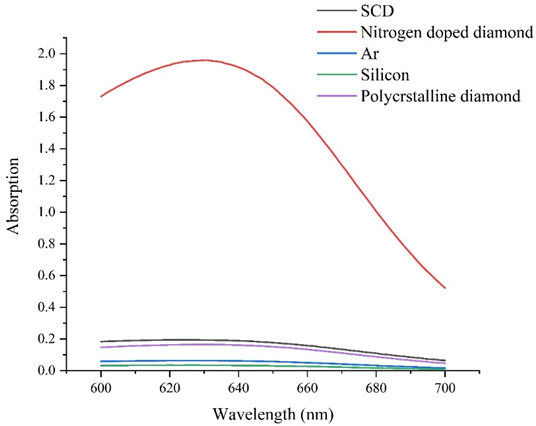


**Figure S6.** Expriments with differernt samples and controls. They are nitrogen doped diamond (called experiment NDD), polycrystalline diamond (PD), single-crystalline diamond (SCD), Ar gas instead N_2_, silicon, respectively. The curve of ammonia absorption solution measured at 635 nm by ultraviolet-visible spectrophotometer after 24 h. The ammonia production rate is calculated by the ratio of the absorbance to the standard curve, and calculated by the formula.


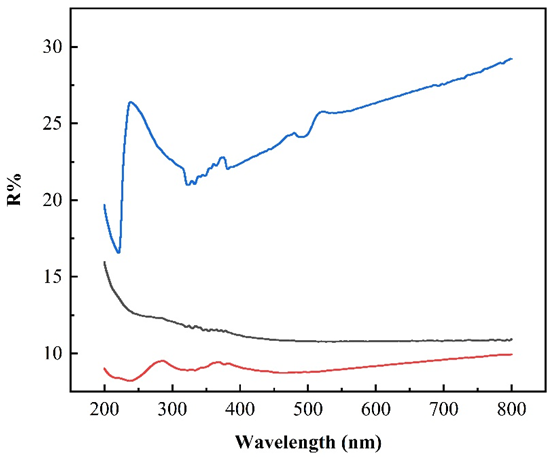


**Figure S7.** Diffuse reflectance spectroscopy (DRS) of nitrogen doped diamond film, polycrystalline diamond film and single crystal diamond film. we measure the optical absorption via transforming UV−vis diffuse reflectance spectroscopy by using an integrating sphere, since the incorporation of nitrogen impurities into the diamond film is accompanied by changes in both optical absorption and scattering from the film.


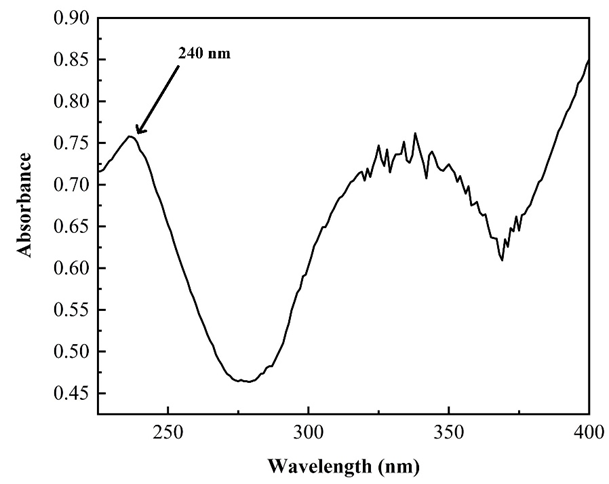


**Figure S8.** absorbance spectra of silicon for contrast, the peak on 240 nm corresponds to a direct (momentum-conserving) optical transition of bulk silicon.

**Table S1.** Linear regression curve fitting by the method of least square.

| **A** | **B1** | **B2** | **B3** |
| --- | --- | --- | --- |
| Equation | Y= a+b*x | | |
| Plot | Nitrogen doped diamond | Polycrystalline diamond | SCD |
| Weight | No Weighting | | |
| Intercept | 39.43107 ± 21.64763 | 10.193 ± 2.81603 | 4.99071 ± 2.71031 |
| Slope | 6.27279 ± 1.48589 | 3.16951 ± 0.19329 | 2.53019 ± 0.18604 |
| Residual Sum of Squares | 1513.4938 | 25.61139 | 23.72449 |
| Pearson’s r | 0.94821 | 0.9963 | 0.99464 |
| R-Square | 0.8991 | 0.99262 | 0.9893 |
| Adj. R-Square | 0.84865 | 0.98892 | 0.98396 |

| 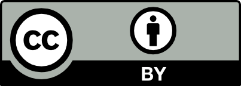 | © 2020 by the authors. Submitted for possible open access publication under the terms and conditions of the Creative Commons Attribution (CC BY) license (http://creativecommons.org/licenses/by/4.0/). |
| --- | --- |
